# Targeting DNA methylation: molecular and in vivo evidence for quinoa and chia nanocapsules as epigenetic modulators in DMBA-treated rats

**DOI:** 10.1038/s41538-026-00946-w

**Published:** 2026-07-03

**Authors:** Dalia M. Mabrouk, Mohamed M. Abd Elrahman, Tamer K. Khatab, Aida I. El makawy

**Affiliations:** 1 Cell Biology Department, Biotechnology Research Institute, National Research Centre, Giza, Egypt; 2 Organometallic and Organometalloid Chemistry Department, National Research Centre, Giza, Egypt

**Keywords:** Biochemistry, Biological techniques, Biotechnology, Cancer, Chemical biology, Drug discovery, Molecular biology

## Abstract

This study investigated the modulating role of quinoa and chia nanocapsules on the DMBA-induced DNA methylation in rats. Theoretical docking was conducted to assess the binding affinities of selected phytoconstituents from quinoa and chia against the active sites of DNMT1 and TET2. An in vivo study was conducted to determine the effect of nanocapsules on DMBA-induced LINE-1 DNA methylation patterns. Molecular docking suggested that all selected compounds have high binding affinity for DNMT1, with linoleic acid being the top-rate TET2 inhibitor. The in vivo study demonstrated that quinoa nanocapsules can potentially reverse the DMBA-induced LINE-1 methylation pattern in the liver and kidney. Chia nanocapsules did not change the methylation status in the kidney; however, they partially modulated it in the liver. Our findings show that DMBA-induced LINE-1 DNA methylation in the kidney and liver is reversible via quinoa nanocapsules, and is likely driven by their active phytochemicals.

## Introduction

A person’s lifestyle and prolonged exposure to xenobiotics, such as industrial chemicals, pesticides, detergents, organic solvents, and polycyclic aromatic hydrocarbons (PAHs), can alter how genes function and modify the epigenome, making them more susceptible to disease^1^. PAHs exhibit a wide range of biological toxicity in terrestrial and aquatic environments^2^. One of the most significant PAHs is 7,12-dimethylbenz[a]anthracene (DMBA), a harmful substance found in exhaust fumes, tobacco smoke, and burned food. DMBA can result in significant behavioral dysfunctions and physiological abnormalities, such as cancer, aging, and hypertension^3^. It is a carcinogen that causes DNA methylation, genetic abnormalities, and DNA damage, leading to cancer^4^. DMBA induces oxidative alterations of DNA bases, which have mutagenic and carcinogenic consequences, and forms DNA adducts by creating strand breaks and increasing oxidative stress in cells during metabolism^3,5^.

In recent years, there has been significant interest in elucidating the mechanisms of epigenetics and their role in the progression of human diseases. DNA methylation is an epigenetic mechanism that involves attaching a methyl group to a cytosine residue within CpG dinucleotides, which are common in gene promoter regions. The main components in the machinery driving methylation and demethylation include DNA methyltransferases (DNMTs) and ten-eleven translocations (TETs) enzymes. Numerous studies have emphasized the impact of DNA methylation alterations on the detrimental health effects of exposure to environmental toxicants^6^. The quantification of DNA methylation levels in repetitive elements, such as long interspersed nucleotide elements (LINEs), can indicate overall DNA methylation^7^. Due to the reversible nature of epigenetic modifications, studies on them may identify new targets or markers for treatment. This could lead to the development of innovative approaches to illness prevention or treatment^8^.

The traditional view of nutrition, which focuses on nutrient accessibility, is now shifting towards a greater appreciation of the bioactive potential of nutrients. This shift has led to the need for customized therapies that incorporate precision medicine and complementary nutrition, according to molecular and metabolic changes. By using life sciences methods to characterize food components with high nutritional value and biological activity, novel, high-value products can be created to improve health^9^. Quinoa and chia are considered superfoods due to their bioactive compounds, minerals, vitamins, and antioxidants, such as myricetin, quercetin, chlorogenic acid, and kaempferol, offering numerous nutritional benefits, such as antioxidant, antidiabetic, antitumor, antihypertensive, and antimicrobial properties, and serving as functional food additives^10–14^.

Chia is a member of the Lamiaceae family, known by its scientific name, Salvia Hispanica L., and is a major vegetable source of alpha-linolenic acid (ALA), making up more than 60% of all fatty acids. Chia is well-known for its high fiber content, abundant antioxidants, and rich omega-3 fatty acids. Chia seeds are characterized by their oil content, ranging from 25 to 30%, and consist of 27.40% omega-6 linolenic acid and 11.96% omega-3 alpha-linolenic acid (ALA), which are essential fatty acids for health^15^. Chia seeds contain ~15–25% protein, 30–33% fat, 26–41% carbohydrates, 18–30% fiber, and around 4–5% ash content^16,17^. Due to its nutritional value and potential health benefits, chia has been the subject of extensive global research in recent decades. It has been hypothesized that consuming chia seeds could help prevent organ failures related to the disease by protecting against lipid accumulation, oxidative stress, and fibrosis in the tissues^18^.

Quinoa is a highly nutritious food with a low glycemic index (GI) and is rich in protein, unsaturated fatty acids, gluten-free content, and essential amino acids. Additionally, quinoa has antibacterial, anti-inflammatory, and antioxidant properties, making it a valuable functional food. Its antioxidant capabilities, attributed to phenolic and flavonoid components, help protect organisms from oxidative stress and free radicals, thus promoting overall health^19^. Quinoa also contains a high concentration of secondary metabolites with antimicrobial, anti-diabetic, anti-inflammatory, and immunomodulatory properties, such as steroids, flavonoids, and triterpene saponins^20^. Moreover, quinoa is a rich source of vitamins B2, B6, and B9, as well as minerals like magnesium, copper, calcium, phosphorus, potassium, and iron, all of which are essential for various biological activities that support human health^21,22^.

Our Previous research demonstrated that chia and quinoa nanocapsules could improve DMBA-induced alterations in liver and kidney function markers^23^. The present study aims to investigate DMBA-induced methylation alterations in the liver and kidney tissues and to determine the extent to which chia and quinoa nanocapsules can modulate methylation changes caused by DMBA. Molecular docking is a tool predicting the preferred orientation of a ligand in the binding site of a target protein, enabling assessment of binding affinity and potential bioactivity. So, an in-silico analysis was conducted to assess the role of chia and quinoa bioactive phytochemical modulators via DNAMT1 and Tet2.

## Results

### Molecular docking study of identified compounds

Chia and quinoa contain high levels of antioxidants, including kaempferol, quercetin, myricetin, chlorogenic acid, α-tocopherol, γ-tocopherol, and linoleic acid. The ligands used in this study were labeled as L1–L7 for simplicity. Each code corresponds to a specific chemical compound, as detailed in Table 1. These compounds were selected as ligands (L1–L7) and nominated for the docking study to determine their binding to the two proposed enzymes (DNMT1, TET2) and to gain further insights to confirm their observed modulator activities against DNA methylation in the in vivo study. The binding process between the ligands and the protein affinity results in lower E-score values. From the data obtained in Table 1, it was clear that all the ligands under study (L1–L7) exhibit strong binding affinity ranging between (−6.36 to −8.36 kcal/mol) and electrostatic bond distance ranging between (2.85 and 4.03 nm) compared to S-adenosyl-L-homocysteine (−7.39 kcal/mol) as a reference ligand when binding with the enzyme DNMT1. The protein’s active site amino acid residues that act as hydrogen donors are Arg1310, Glu1266, Ser1146, and Glu1168, and those that act as hydrogen acceptors are Gly1223, Cys1148, Asn1578, Gly1150, Leu1151, Val1580, Cys1191, and Phe1145. In the case of the TET2 enzyme, the binding affinity of the ligands (L1–L7) ranges between 0.58 and −6.24 kcal/mol. The ligand L7 showed a higher binding affinity (−6.24 kcal/mol) with an electrostatic bond distance range of 1.92 and 2.93 nm compared to the reference ligand N-Oxalylglycine (−7.1 kcal/mol). The active site amino acid residues acting as hydrogen donors are His141 and Ser1898, and Arg1261 and Arg1896 act as hydrogen acceptors.

**Table 1:** Energy score values for the targeted ligands binding with DNMT 1 and TET 2

Ligand number	Ligand structure	E-score Kcal/mol (DNMT 1)	E-score Kcal/mol (TET 2)
L1		−6.36	−2.05
L2		−6.94	−1.88
L3		−6.98	−2.19
L4		−6.89	−2.26
L5		−8.15	0.58
L6		−8.36	−1.76
L7		−7.78	−6.24
Ref. for DNMT1	S-Adenosyl-L-Homocysteine	−7.39	—
Ref. for TET2	N-Oxalylglycine	—	−7.10

**Fig. 1:**
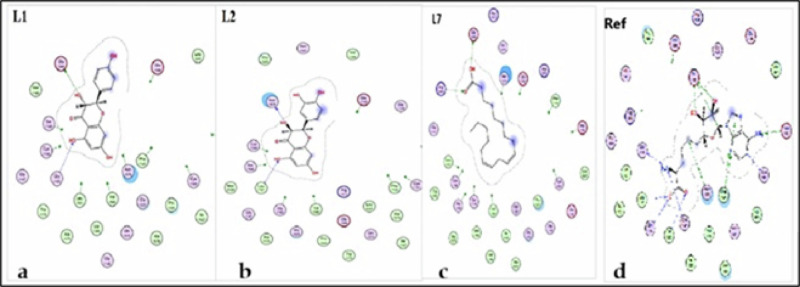
The 2D photos of three selected ligands. **a** L1; **b** L2; **c** L3; **d** S-Adenosyl-L-Homocysteine as ref. and the active site in DNMT1.

**Fig. 2:**
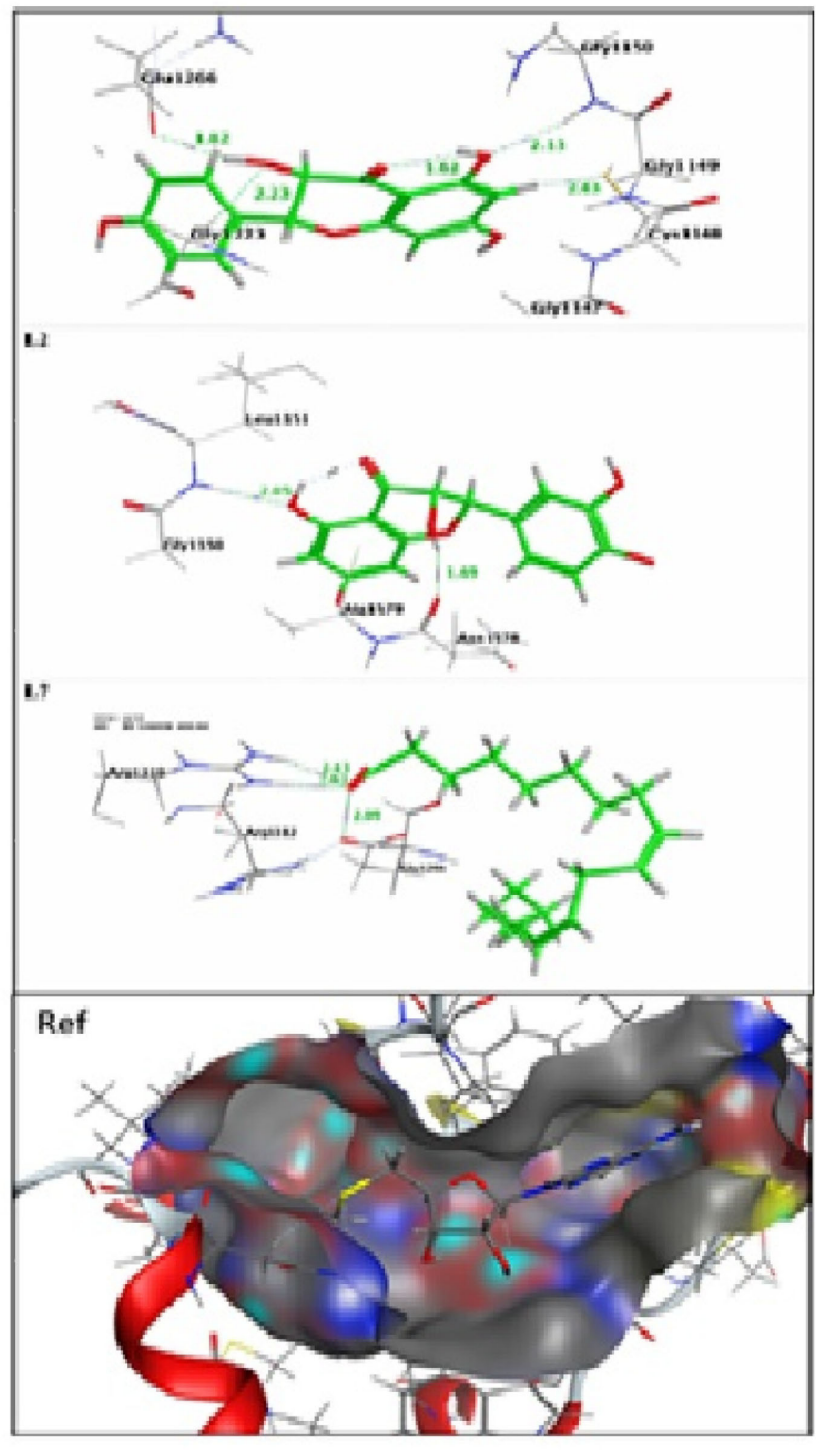
Molecular docking analysis showing the 3D of three selected ligands L1, L2 and L7 and S-Adenosyl-L-Homocysteine with active site in DNMT1.

**Fig. 3:**
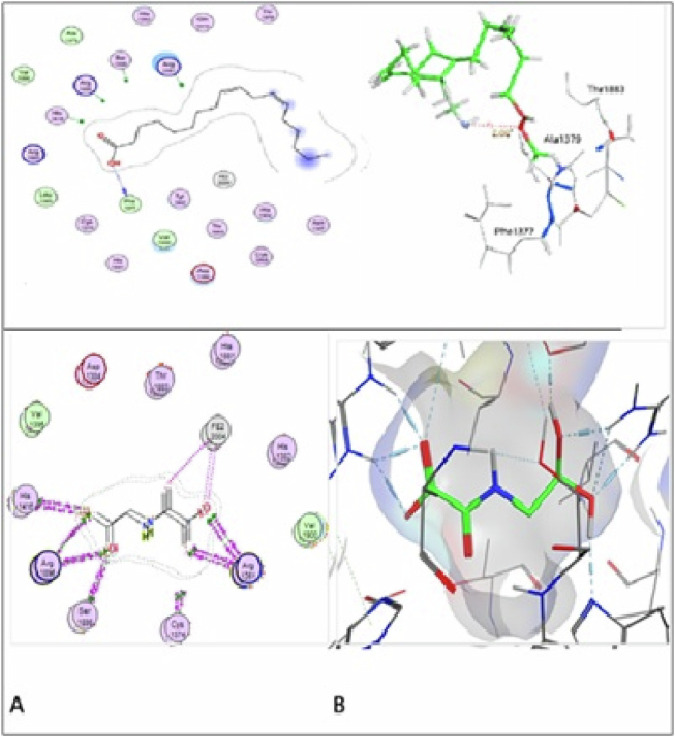
Photos of Linoleic acid (L7) and N-Oxalylglycine active sites Tet2 enzyme. **A** Represents the 2D and **B** the 3D.

Figure 1 presents two-dimensional photos of the interaction between ligands L1, L2, and L7 with the DNMT1 enzyme. The photos depict the geometric interaction between the ligands and all active site amino acid residues in the DNMT1 enzyme. The 3D photos in Fig. 2 illustrate that kaempferol (L1) forms four H-bonds with the cytosine binding site of DNMT1. The binding sites are Glu1266 (bond distance 1.82 nm), Gly1223 (bond distance 2.23 nm), Gly1150 (bond distance 2.13 nm), and Cys1148 (bond distance 2.61 nm). Quercetin (L2) formed three H-bonds with DNMT1 at the binding sites of Gly1150 (bond distance 2.05 nm) and Asn1578 (bond distance 1.69 nm) and a π-π stacking bond with Ala1579. The linoleic acid (L7) binding model is characterized by three H-bonds, two of them binding with the side chain of Arg1310 (bond distance 2.03 and 2.43 nm) and the other with Glu1266 (bond distance 2.09 nm). Regarding the geometric interaction between ligands and the active site amino acid residues in the TET2 enzyme, linoleic acid (L7) exhibited a high binding affinity (−6.24 kcal/mol). Figure 3A displays two-dimensional images of the interaction between the linoleic acid ligand (L7) and the TET2 enzyme. Additionally, Fig. 3B illustrates the binding process through 3D images, showing the **L7**-TET2 complex with a hydrogen bond between the linoleic acid carboxylic group and Phe1377 (bond distance 2.02 nm).

### Nanocapsulation formulation

The morphology and particle size of the nanocapsules were examined using transmission electron microscopy (TEM). The chia oil nanocapsules appeared polygonal, with mean particle sizes ranging from 10 to 35 nm. On the other hand, the quinoa capsules were spherical, with a mean size range of 5–30 nm.

### Determination of LINE-1 DNA methylation

**Fig. 4:**
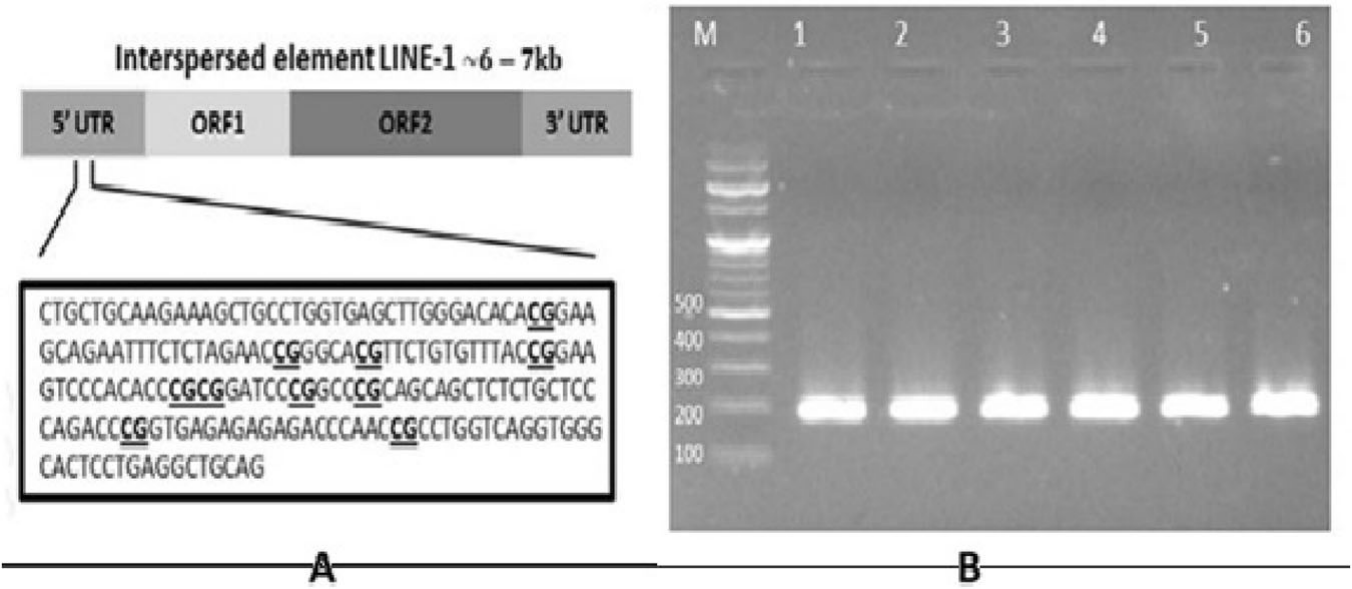
Plate showing. **A** Schematic diagram of LINE-1 showing a 187 bp fragment (base pairs 86–263) in the promoter region of LINE-1, with 10 CpG sites. **B** Representative agarose gel electrophoresis of bisulfite-treated DNA amplified across the promoter region of the LINE-1 gene from liver and kidney tissue. M: DNA marker, Lane 1 shows negative control, Lanes 2–9 show PCR products of the amplified LINE-1fragment.

**Fig. 5:**
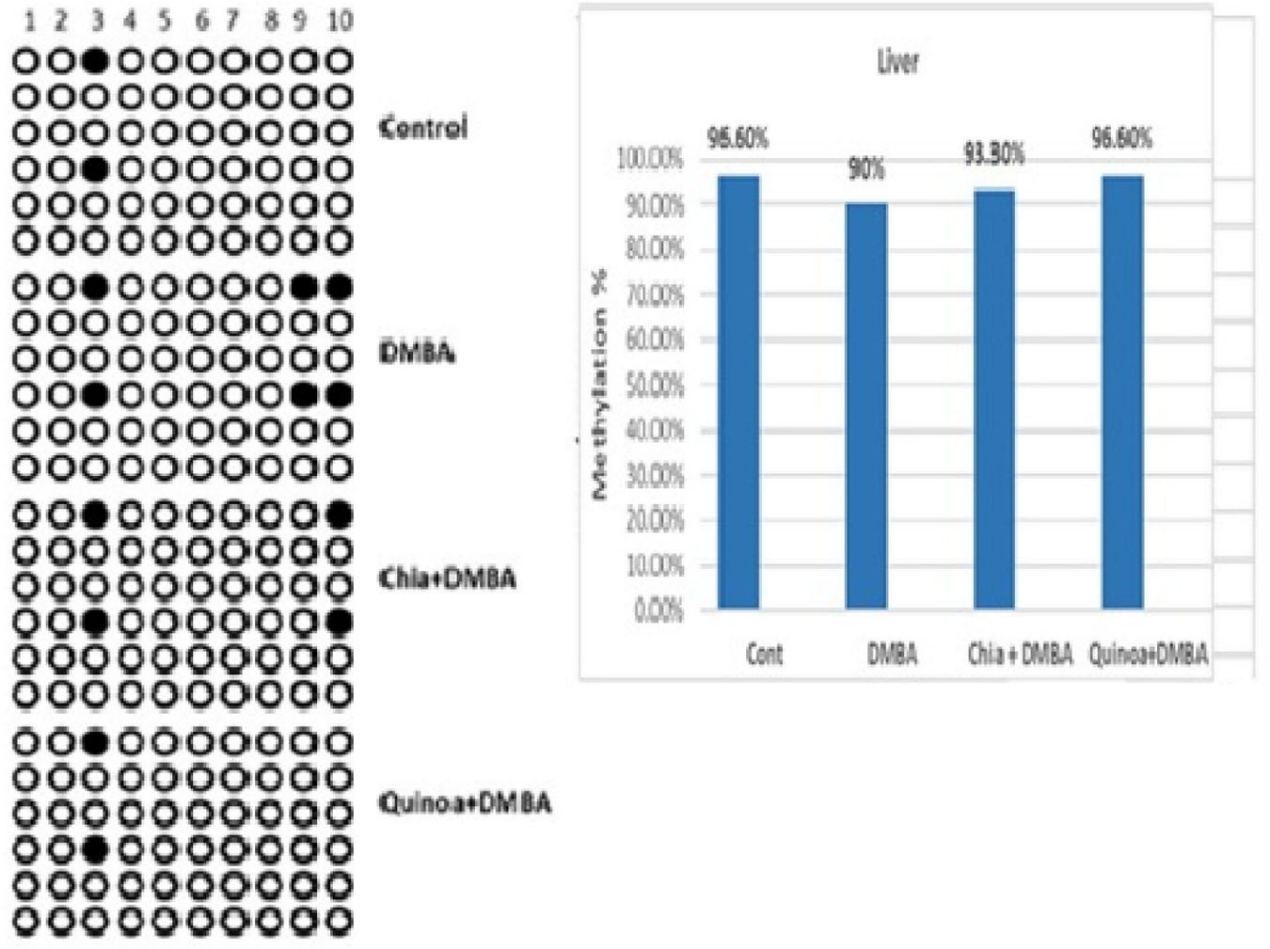
LINE-1 DNA methylation levels in liver tissue treated with DMBA, DMBA + Chia nanocapsules, and DMBA + Quinoa nanocapsules. Black and white circles represent unmethylated and methylated cytosines, respectively. **b** The histogram of the methylation level.

**Fig. 6:**
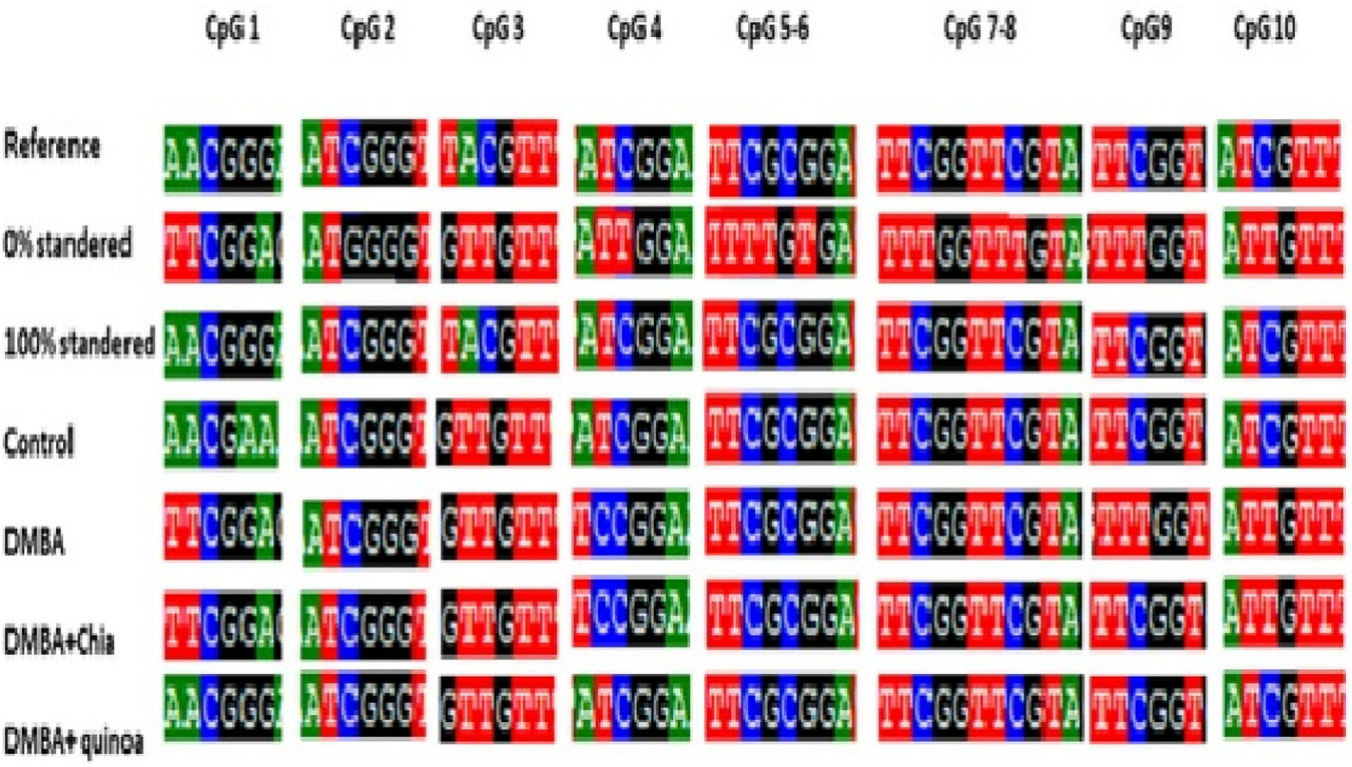
Diagram displaying the results of bisulfite sequencing of liver tissue samples from 0% standard, 100% standard, control, DMBA, DMBA + chia nanocapsules and DMBA +quinoa nanocapsules compared to the reference sequence for LINE-1.

**Fig. 7:**
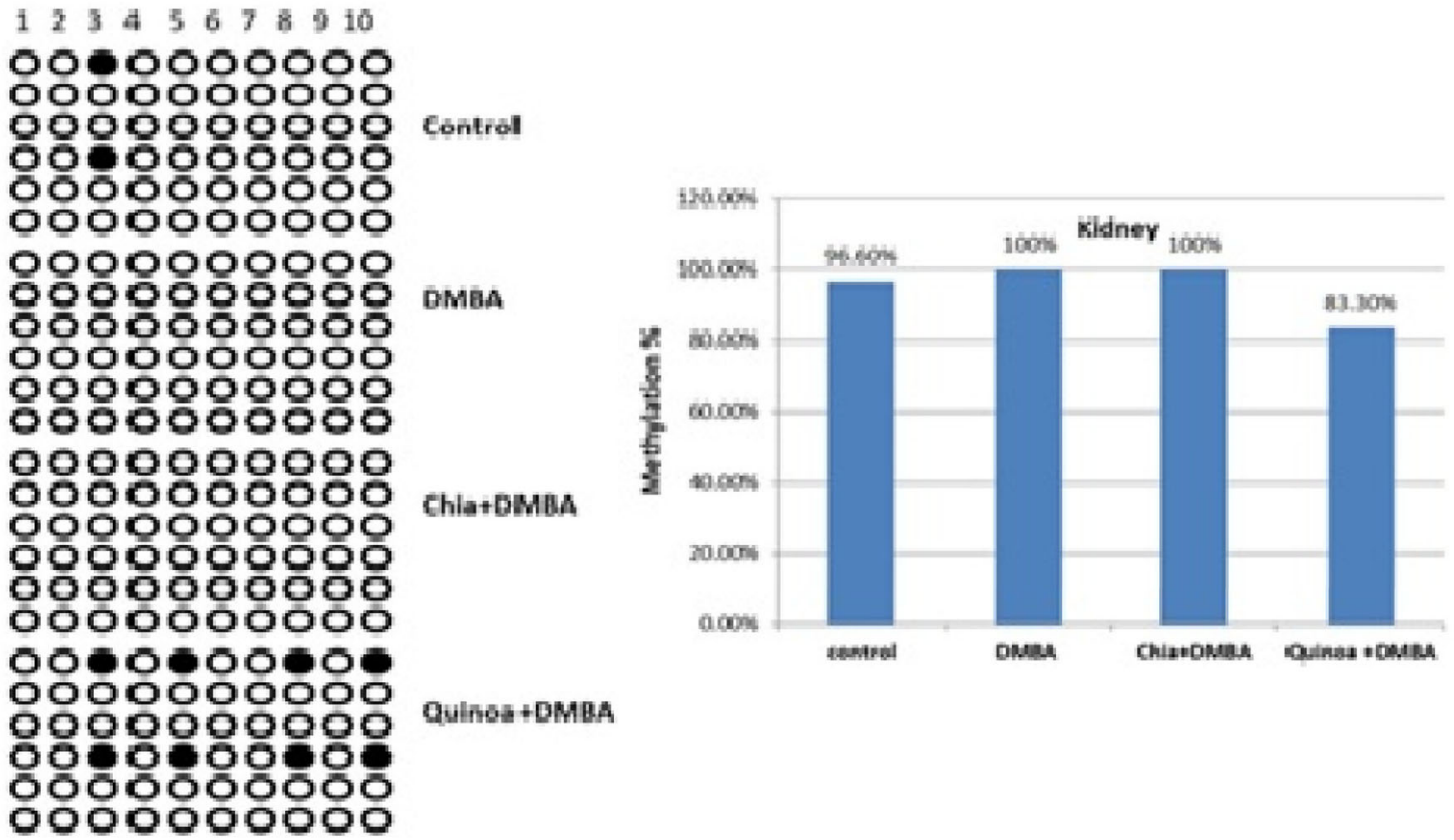
LINE-1 DNA methylation levels in kidney tissue treated with DMBA, Chia, and Quinoa nanocapsules. Black and white circles represent unmethylated and methylated cytosines, respectively. **b** The histogram of the methylation level.

**Fig. 8:**
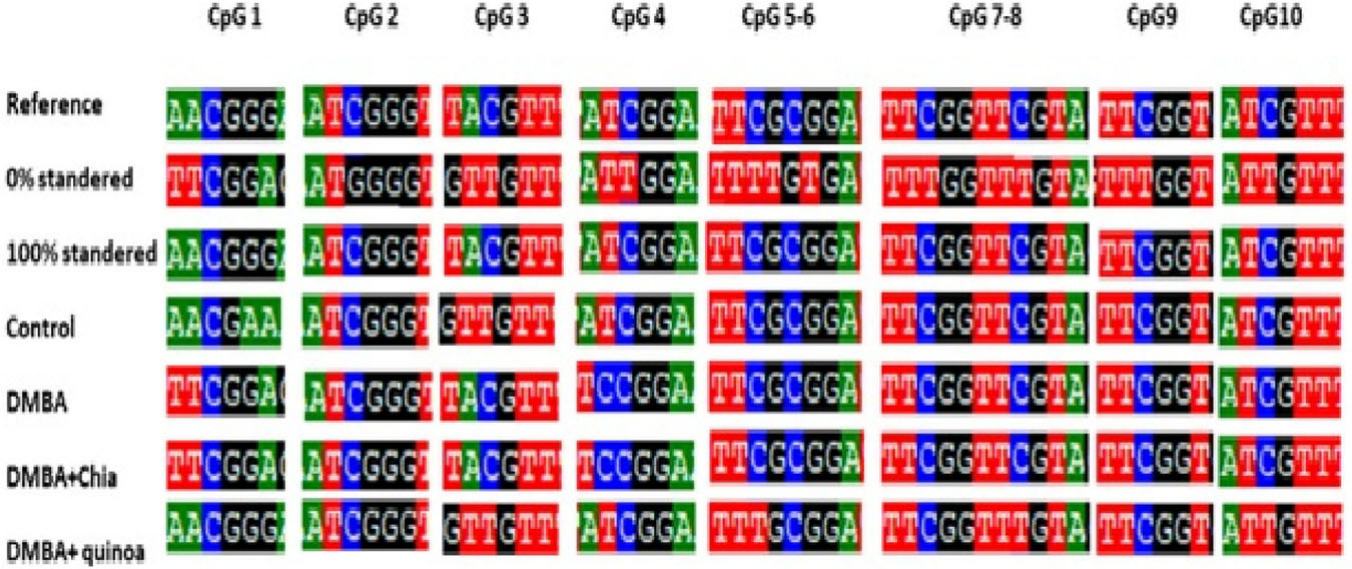
Diagram displaying the results of bisulfite sequencing of kidney tissue samples from 0% standard, 100% standard, control, DMBA, DMBA + chia nanocapsules, and DMBA + quinoa nanocapsules compared to the reference sequence for LINE-1.

Figure 4A represents the 187 bp amplified fragment in the promoter region of LINE-1, displaying 10 CpG sites. Figure 4B shows a representative agarose gel electrophoresis of bisulfite-treated DNA amplified across the promoter region of the LINE-1 gene from liver and kidney tissue. As shown in Figs. 5 and 6, DMBA induced LINE-1 DNA hypomethylation in the liver compared to the untreated control. Treatment with chia nanocapsules could decrease the hypomethylating effect of DMBA moderately; quinoa showed the potential to ameliorate the DMBA hypomethylating effect completely. In contrast, DMBA induced LINE-1 DNA hypermethylation in the kidney. Chia nanocapsules did not result in any changes compared the DMBA group, while quinoa showed the potential to alleviate the hypomethylating effect of DMBA completely. Interestingly, the sequencing indicated that quinoa nanocapsules can restore the methylation status of the same CpG sites that DMBA alters in the liver and kidney (Figs. 7 and 8).

## Discussion

DMBA is an environmental pollutant that can cause several diseases. Research shows that when DMBA is given to rats, it can lead to damage to their livers and kidneys. This damage is characterized by elevated levels of liver and kidney function, oxidative stress, inflammation, and histopathological alterations, including fibrosis^24–26^. Exposure to environmental factors, including pollutants, can change the epigenome and lead to adverse health effects. Pollutants such as DMBA have been linked to epigenetic changes, including DNA methylation^27,28^.

Trends toward LINE-1 hypomethylation were observed in response to DMBA treatment in all tissue types in previous studies^3,28^. Unexpectedly, our findings confirmed that LINE-1 methylation status differs between the liver and kidney tissues after DMBA treatment. Results showed that DMBA induced LINE-1 DNA hypomethylation in liver tissue. These results are consistent with previous studies by Szabo et al.^3,28^, who examined the LINE-1 DNA methylation patterns in DMBA-induced hepatic injury using HRM analysis. Our results are also consistent with the LINE-1 methylation status observed in liver injury in animal models.Tryndyak et al.^29^ showed that LINE-1 hypomethylation reveals key pathophysiological events in liver injury induced by a methyl-deficient diet. While not focusing on LINE-1 DNA methylation, studies have identified DNA hypomethylation at specific loci. Liver injuries induced by xenobiotics such as arsenic and carbon tetrachloride caused a marked decrease in DNA methylation levels^4,30^. A similar change is also observed in *K. pneumonia-*induced liver damage^31–33^. Additionally, strong correlations between LINE-1 hypomethylation and liver damage have been demonstrated in clinical research^31,34^.

Changes in the expression and/or activity of DNMTs have been suggested as one of the potential causes of DNA hypomethylation. Liver injury-induced DNA hypomethylation, reported in several previous studies, has been attributed to the reduced expression of the genes DNMT1, DNMT3b, and DNMT3L^4,35–37^. Since the balance between methylation and demethylation processes determines the final state, it is crucial to investigate both DNMTs and TETs when researching global DNA methylation^38^. Recently, Sun et al.^39^ demonstrated that upregulation of TET3 was accompanied by promoter hypomethylation in the liver in mice and patients.

In our study, DMBA treatment caused significant LINE-1 DNA hypermethylation in kidney tissue. Interestingly, it is reported that DMBA treatment induced hypomethylation in kidney tissue^3,28^. One reason for this could be the use of different assays targeting different CpGs within the target sequence^40^. Furthermore, studies reported lower methylation in MS-HRM than calculated by sequencing^19,41^. In addition, DMBA resulted in DNA hypomethylation, caused by inhibition of the DNMT enzyme. Furthermore, DMBA also causes hypomethylation of oncogenes (e.g., *HA-RAS*), and hypermethylation of tumor suppressor genes (e.g., *P53*) by affecting the methylation of CpG islands of DNMT enzymes themselves. Consequently, these can lead to increased cell proliferation and increased carcinogenesis^28^.

Our observation is consistent with previous studies that reported LINE-1 hypermethylation in kidney disease^42^. Additionally, Nascimento Goncalves et al.^43^ reported that preimplantation biopsies from non-ideal kidneys displayed increased methylation levels in LINE-1, as well as high expression levels of DNMT1 and DNMT3B compared to ideal kidneys. Liao et al.^44^ found that higher levels of LINE-1 methylation are positively correlated with an increased risk of renal cancer. While not focusing on LINE-1 DNA methylation, emerging evidence from in vitro and in vivo studies on various loci has supported our findings that DNA hypermethylation is associated with kidney injury. Sun et al.^45^ suggested that the treatment of human kidney (HK2) cells with indoxyl sulfate or p-cresyl sulfate increased CpG hypermethylation of the Klotho gene and induced the expression of DNA methyltransferases 1, 3a, and 3b isoforms. Zeisberg et al.^46^ detected a significant increase in DNA methylation in fibrotic kidneys after folic acid-induced nephropathy. Expression of DNMT1 was reported to be markedly increased in diabetic nephropathy^47^. Recently, Xiao et al.^48^ suggested that fibrosis of the mouse kidney was associated with global DNA hypermethylation and increased the expression of DNA methyltransferases, which was alleviated by the DNA methyltransferase inhibitor 5-Aza-2′-deoxycytidine. Moreover, studies on TET1 and TET2 knockout mice have suggested that TET1 and TET2 protect against kidney injury by repressing inflammatory responses, ROS, inflammatory markers, and fibrosis^48–50^. Koh et al.^51^ suggested that inhibiting DNA methylation restores the disrupted balance between pro-inflammatory and anti-inflammatory pathways, alleviating renal inflammation and fibrosis.

A growing body of research has demonstrated the involvement of epigenetic dysregulations in a broad spectrum of diseases. The reversible nature of epigenetic alterations, in terms of activating or inhibiting implicated enzymes, may make them attractive targets and practical tools for understanding biological and cellular processes. Natural products from various sources, such as plants, can influence epigenetic modifications^52^. In this study, we investigated the impact of chia and quinoa nanocapsules on DMBA-induced LINE-1 methylation patterns. While DMBA caused hypomethylation in the liver and hypermethylation in the kidney, quinoa nanocapsules were able to modulate both patterns. Treatment with chia nanocapsules did not change the methylation status in the kidney; however, it partially increases hypermethylation in the liver. Findings from our investigators and others have shown that quinoa and chia seeds contain bioactive compounds with medicinal properties that can help combat various diseases, including cancer. These compounds include phytosterols, saponins, phenolic compounds, phytoecdysteroids, polysaccharides, and betalains. They are known to be anti-apoptotic, anti-inflammatory, and anti-oxidant^10,21,22^.

Emerging evidence suggests that these bioactive compounds exert effects by altering gene expression through epigenetic markers, such as DNA methylation. The in-silico docking study of the selected compounds into DNMT1 (pdb: 4WXX) and TET2 (pdb: 4NM6) target proteins suggested that the observed DNA methylation modulating effect of quinoa through inhibition of DNMT1 or TET2 enzymes could be attributed to kaempferol (L1), quercetin (L2), myricetin (L3), chlorogenic acid (L4), α, γ-tocopherol (L5 & L6), and linoleic acid (L7). These compounds have high binding affinity to the DNMT1, and linoleic acid showed the highest docking score for TET2. Previous studies have shown that various phytochemicals found in quinoa and chia modulate DNA methylation by either inducing or repressing DNMT expression. Gamma-tocopherol was found to reduce DNMT1, DNMT3a, and DNMT3b protein levels and reverse promoter hypermethylation in mice’s prostate tissues. Kaempferol can affect DNA methylation by inhibiting DNMT3B protein levels in bladder cancer^53^. Investigations by Kedhari Sundaram et al.^54^ revealed that quercetin mediates the decrease in global methylation levels of HeLa cells. It also decreased the total DNMTs in the livers of nickel-treated mice. Myricetin was found to inhibit DNMT1-mediated DNA methylation^55^. Romagnolo et al.^56^ suggested that linoleic acid increased the risk of inflammation and colon cancer through DNA methylation. Alpha-tocopherol (Vitamin E) has also been shown to reduce DNMT1 methylation and expression^57^. Chlorogenic acid has hypomethylating effects by inhibiting DNMTs activity in meningioma cells^58^.

Interestingly, the same gene (LINE-1 here) undergoes hypomethylation in the liver and hypermethylation in the kidney after treatment with the quinoa nanocapsules. This suggests that different compounds may have different outcomes on the methylation state of a specific DNA locus. This is supported by in silico results, which indicate that selected compounds found in quinoa and chia have the potential to inhibit both DNMT1 and TET2.

This study has some limitations that should be acknowledged. First, the sample size was relatively small. Second, the study relied on LINE-1 methylation as a surrogate marker of global DNA methylation, which may not fully reflect gene-specific methylation changes. Third, the expression and activity levels of key enzymes involved in DNA methylation and demethylation (e.g., DNMTs and TETs) were not directly measured. Finally, bisulfite sequencing was performed once per sample without technical replicates.

Interestingly, our results suggest different methylation patterns in liver and kidney tissues in response to DMBA. Quinoa nanoparticles have the potential to reverse the DMBA-induced LINE-1 methylation pattern in various tissues. Further work with a larger sample size is warranted to confirm the results. Additionally, further research is needed to investigate the expression levels of DNMTs and TETs to explore the mechanisms of LINE-1 methylation modulation. Future studies should include larger sample sizes to validate and strengthen the current findings. In addition, assessing the expression and activity levels of DNA methylation-related enzymes, such as DNMTs and TETs, would provide deeper mechanistic insights into the observed epigenetic modulation. Expanding the analysis to include gene-specific methylation and genome-wide approaches would further clarify the epigenetic impact of quinoa and chia bioactive compounds. Moreover, incorporating technical replicates in bisulfite sequencing assays is recommended to enhance the reproducibility and reliability of the data.

## Materials and methods

### Chemicals

The 7,12-dimethylbenz(a)anthracene (DMBA) was purchased from Sigma-Aldrich (St. Louis, MO, USA). The bisulfite conversion and PCR purification kits were purchased from Thermo Fisher Scientific, UK. All other chemicals used throughout the experiments were of the highest analytical grade.

### Molecular docking

Seven phytochemicals, namely kaempferol, quercetin, myricetin, chlorogenic acid, α, γ–tocopherol, and linoleic acid, were selected as desired ligands for this study. The selected phytochemicals were chosen based on published compositional analyses of quinoa and chia seeds, which demonstrate their abundance and biological relevance, particularly their reported roles in antioxidant activity and epigenetic modulation^10–15^.

The docking steps were using MOE 2015.10 to measure the degree of binding between ligands and enzymes based on established standards^58,59^. The DNMT1 and TET2 proteins were obtained from the Protein Data Bank (PDB) through the mdb file (PDB code: 4WXX and 4NM6) and subjected to this protocol. The docking study begins by downloading the 3D structure of the protein (Protein Data Bank PDB: 4WXX and 4NM6 for DNMT1 and TET2, respectively)^60,61^ in MOL2 format. The docking process involved preparation of the target enzyme by removing water molecules and adding hydrogen atoms, followed by energy minimization. Subsequently, a direct (site-specific) docking protocol was performed, and the grid box was defined around the known active site. The selected ligands in Table 1 were docked into this region. The binding affinity was evaluated using docking scores (kcal/mol), which estimate the strength of interaction between the ligand and the enzyme. These scores are based on the predicted geometry of the protein–ligand complex, including hydrogen bonding, hydrophobic interactions, and other stabilizing forces within the active site^62,63^.

### Preparation and characterization of nanocapsules

According to our previous studies^15,64^, we prepared and characterized sodium alginate nanocapsules containing chia or quinoa. Oil and sodium alginate (Sigma-Aldrich) were emulsified using an aqueous phase containing 3% (w/v) alginate and 30 g of oil. The mixture was vigorously stirred at room temperature using a mechanical stirrer (Greave Mixer, England) until a creamy emulsion. The resulting emulsion was then sonicated for 30 min using an ultrasonic cleaning bath (WUC-DO3H, 290 W, 60 Hz), followed by high-energy ultrasonication for 3 min with a probe sonicator (VCX750, 750 W, 20 kHz). To promote equilibration and nanoparticle stabilization, a calcium chloride solution (CaCl₂:alginate ratio 2:10) was added, and the mixture was further sonicated under the same conditions, yielding a stable oil-in-water nanoemulsion.

### Animals

Forty female Sprague-Dawley rats, aged 6–8 weeks and weighing 150–180 g, were obtained from the National Research Centre’s animal facility in Egypt. They were kept in controlled conditions with regulated humidity, ventilation, temperature (22 ± 1 °C), and a light-dark cycle (7:00–19:00 h). All animal procedures were approved by the National Research Centre ethical committee (registration number 19164) and National Institutes of Health’s Guide for the Care and Use of Laboratory Animals (NIH Publications No. 8023, amended 1978). The rats were divided into four groups, each of ten rats, as follows: Group 1: animals received saline. Group 2: rats were given a single subcutaneous injection of 80 mg/kg DMBA in 0.5 ml of corn oil. Group 3: Animals were injected with DMBA and then orally administered 200 mg/kg/day of quinoa oil nanocapsules for a month. Group 4: Animals were injected with DMBA and then orally given Chia oil nanocapsules (200 mg/kg/day) for a month.

### Anesthesia and tissue collection

The rats were anesthetized through intraperitoneal (IP) injections of xylazine (40 mg/kg b.w.) and ketamine (4 mg/kg b.w.). The animals were sacrificed, and the liver and kidneys were dissected for future analysis.

### Genomic DNA extraction

The liver and kidney tissue were ground in liquid nitrogen, and the genomic DNA was extracted using the Genomic DNA Purification Kit (ThermoFisher, UK) according to the manufacturer’s instructions. DNA integrity was assessed by running it on a 2% agarose gel. DNA purity and concentration were assessed using the NanoDrop^TM^ 1000 Spectrophotometer (Thermo Fisher Scientific, USA).

### Standard samples

For the synthesis of methylated DNA standard (100%), purified genomic DNA was amplified using the REPLI-g® Mini Kit (QIAGEN) with 5 μl of template DNA, following the manufacturer’s instructions. To synthesize an unmethylated DNA standard (0%), a portion of the unmethylated standard prepared previously will be methylated using CpG Methyltransferase (M.SssI) from Thermo Fisher Scientific, following the manufacturer’s protocol.

### Bisulfite treatment

A DNA sample (20 μL) containing 200–500 ng of purified genomic DNA was treated with the EpiJET Bisulfite Conversion Kit (Thermo Fisher Scientific, UK). During treatment, unmethylated cytosine is converted into uracil, while methylated cytosine remains unchanged and stays as cytosine.

### PCR and direct bisulfite sequencing

The primers used for analyzing LINE-1 methylation were as follows: Forward, TTGTTGTAAGAAAGTTGTTTGGTGA; reverse, CTACAACCTCAAAAATACCCACCTA. These primers targeted 10 CpGs in an amplicon of 187 bp and were initially described by Park et al.^19^. PCR was conducted in a 30 µL reaction volume, including 15 ng of bisulfite-converted DNA templates, 10 pmol of the forward primer, 10 pmol of the reverse primer, and 15 µL of TOPsimple™ PCR DyeMIX-nTaq (Enzynomics, Korea). The PCR reaction conditions were as follows: 95 °C for 5 min, followed by 35 cycles of 95 °C for 30 sec, 56 °C for 30 sec, and 72 °C for 30 s, with a final extension at 72 °C for 5 min. The PCR products were then analyzed on a 1.5% agarose gel to confirm specificity. Subsequently, the PCR products were purified using the GeneJET PCR Purification Kit (Thermo Fisher Scientific, UK) and sent to Macrogen Company for sequencing.

### Data and statistical analysis

The sequencing results were aligned with the original (unconverted) sequence in GenBank and analyzed using QUMA (Quantification Tool for Methylation Analysis; http://quma.cdb.riken.jp/) to determine the positions and percentages of methylated cytosine residues. The percent methylation was calculated by dividing the number of methylated CpG sites by the total number of true CpG sites^65^. The Mann–Whitney U test was used to determine statistical significance.

## Data Availability

Data is provided within the manuscript file.

## References

[1] Mazambani, S., Morris, M. & Cheriyath, V. Epigenome-modulated xenobiotic detoxification pathways control DMBA-induced breast cancer in agouti Avy/a mice. *Epigenetics* **14**, 708–720 (2019). 31070092 10.1080/15592294.2019.1610306 PMC6557611

[2] Adeniji, A. O., Okoh, O. O. & Okoh, A. I. Levels of polycyclic aromatic hydrocarbons in the water and sediment of Buffalo River Estuary, South Africa and their health risk assessment. *Arch. Environ. Contam. Toxicol.* **76**, 657–669 (2019). 30879120 10.1007/s00244-019-00617-w PMC6469821

[3] Szabo, L. et al. Olive oil improves while trans fatty acids further aggravate the hypomethylation of LINE-1 retrotransposon DNA in an environmental carcinogen model. *Nutrients* **14**, 908 (2022). 35215560 10.3390/nu14040908 PMC8878525

[4] Zhang, R. et al. Combined methylation and transcriptome analysis of liver injury of nonalcoholic fatty liver disease induced by high alcohol-producing Klebsiella pneumoniae. *Microbiol. Spectr.* **11**, e0532322 (2023). 37022192 10.1128/spectrum.05323-22 PMC10269619

[5] Mumtaz, S. et al. Therapeutic role of garlic and vitamins C and E against toxicity induced by lead on various organs. *Environ. Sci. Pollut. Res. Int.* **27**, 8953–8964 (2020). 32036533 10.1007/s11356-020-07654-2

[6] Valente, A., Vieira, L., Silva, M. J. & Ventura, C. The effect of nanomaterials on DNA methylation: a review. *Nanomaterials* **13**, 1–22 (2023). 10.3390/nano13121880 PMC10305477 37368308

[7] Pappalardo, X. G. & Barra, V. Losing DNA methylation at repetitive elements and breaking bad. *Epigenet. Chromatin* **14**, 1–21 (2021). 10.1186/s13072-021-00400-z PMC8173753 34082816

[8] Wu, Y. L. et al. The relationship between ultraviolet B and DNA methylation in skin cancers. *Int. J. Dermatol. Venereol.* **6**, 157–162 (2023).

[9] Laparra Llopis, J. M., Brown, D. & Saiz, B. Chenopodium quinoa and Salvia hispanica provide immunonutritional agonists to ameliorate hepatocarcinoma severity under a high-fat diet. *Nutrients* **12**, 1946 (2020). 32629893 10.3390/nu12071946 PMC7400258

[10] Agarwal, A. et al. Nutritional and functional new perspectives and potential health benefits of quinoa and chia seeds. *Antioxidants* **12**, 1413 (2023). 37507952 10.3390/antiox12071413 PMC10376479

[11] Marcos Pasero, H., Bojarczuk, A., Haros, C. M. & Laparra Llopis, J. M. Immunonutritional benefits of chenopodium quinoa’s ingredients preventing obesity-derived metabolic imbalances. *Biol. Life Sci. Forum* **17**, 20 (2022).

[12] Mihai, E. et al. In Vitro antioxidant activity determination of a microencapsulated synergic polyphenols-Polysaccharide mixture. *Chem. Proc.* **7**, 31 (2022).

[13] Olmos, E., Roman-Garcia, I., Reguera, M., Mestanza, C. & Fernandez-Garcia, N. An update on the nutritional profiles of quinoa (Chenopodium quinoa Willd.), amaranth (Amaranthus spp.), and chia (Salvia hispanica L.), three key species with the potential to contribute to food security worldwide. *JSFA Rep.* **2**, 591–602 (2022).

[14] Ozón, B., Cotabarren, J., Valicenti, T., Graciela Parisi, M., Obregón, D. avid & Chia expeller, W. A promising source of antioxidant, antihypertensive and antithrombotic peptides produced by enzymatic hydrolysis with Alcalase and Flavourzyme. *Food Chem.* **380**, 132185 (2022). 35093662 10.1016/j.foodchem.2022.132185

[15] El Makawy, A. I. et al. The suppressive role of nanoencapsulated chia oil against DMBA-induced breast cancer through oxidative stress repression and tumor genes expression modulation in rats. *Mol. Biol. Rep.* **49**, 10217–10228 (2022). 36063350 10.1007/s11033-022-07885-1 PMC9618492

[16] Chaudhary, N., Dangi, P., Kumar, R. & Bishnoi, S. Chia seeds—a renewable source as a functional food. *Handbook of Cereals, Pulses, Roots and Tubers* 235–252 (2021).

[17] Onneken, P. Salvia hispanica L. (chia seeds) as brain superfood—how seeds increase intelligence. *J. Neurodegener. Disord*. **2**, 32–34 (2018).

[18] Creus, A., Ingaramo, P., Oliva, M. E. & D’Alessandro, M. E. G. Dietary Salvia hispanica L. seed counteracted kidney failure in experimental metabolic syndrome. *J. Food Nutr. Metab*. **5**, 1–11 (2022).

[19] Park, B. M., Yoon, O. J. & Lee, D. H. Global DNA methylation patterns and gene expression associated with obesity-susceptibility in offspring of pregnant Sprague-Dawley rats exposed to BDE-47 and BDE-209. *Korean J. Clin. Lab. Sci.* **49**, 28–39 (2017).

[20] Yun, S. & Zhang, X. Genome-wide identification, characterization and expression analysis of AGO, DCL, and RDR families in Chenopodium quinoa. *Sci. Rep.* **13**, 1–15 (2023). 36871121 10.1038/s41598-023-30827-1 PMC9985633

[21] Angeli, V. et al. Quinoa (Chenopodium quinoa Willd.): an overview of the potentials of the “golden grain” and socio-economic and environmental aspects of its cultivation and marketization. *Foods* **9**, 216 (2020). 32092899 10.3390/foods9020216 PMC7074363

[22] Kumar, L., Bisen, M., Khan, A., Kumar, P. & Patel, S. K. Role of Matrix Metallo-proteinases in Musculoskeletal Diseases. *Biomedicines* **10**, 2477 (2022). 36289739 10.3390/biomedicines10102477 PMC9598837

[23] El Makawy, A. I. et al. Formulation of quinoa oil-alginate loaded nanoemulsion and its anticancer efficacy as a therapy for chemically induced breast cancer. *Mol. Biol. Rep.* **51**, 705 (2024). 38824214 10.1007/s11033-024-09619-x

[24] Alessandra-Perini, J. et al. Euterpe oleracea extract inhibits tumorigenesis effect of the chemical carcinogen DMBA in breast experimental cancer. *BMC Complement. Altern. Med.* **18**, 1–11 (2018). 29609579 10.1186/s12906-018-2183-z PMC5879811

[25] Hosny, S., Sahyon, H., Youssef, M. & Negm, A. Oleanolic acid suppressed DMBA-induced liver carcinogenesis through induction of mitochondrial-mediated apoptosis and autophagy. *Nutr. Cancer* **73**, 968–982 (2021). 32519911 10.1080/01635581.2020.1776887

[26] Zeweil, M. M. et al. Annona Muricata L. extract restores renal function, oxidative stress, immunohistochemical structure, and gene expression of TNF-α, IL-β1, and CYP2E1 in the kidney of DMBA-intoxicated rats. *Front. Pharmacol.* **15**, 1–16 (2024). 10.3389/fphar.2024.1348145 PMC10867119 38362149

[27] Rubio, K. et al. Nutriepigenomics in environmental-associated oxidative stress. *Antioxidants* **12**, 1–28 (2023). 10.3390/antiox12030771 PMC10045733 36979019

[28] Szabo, L. et al. The effects of flavonoids, green tea polyphenols and coffee on DMBA induced LINE-1 DNA hypomethylation. *PLoS One* **16**, 1–14 (2021). 10.1371/journal.pone.0250157 PMC8057585 33878138

[29] Tryndyak, V. P. et al. Coupling global methylation and gene expression profiles reveal key pathophysiological events in liver injury induced by a methyl-deficient diet. *Mol. Nutr. Food Res.* **55**, 411–418 (2011). 20938992 10.1002/mnfr.201000300

[30] Komatsu, Y. et al. Global analysis of DNA methylation in early-stage liver fibrosis. *BMC Med. Genom.* **5**, 5 (2012). 10.1186/1755-8794-5-5 PMC3295686 22281153

[31] Anwar, S. L. et al. LINE-1 hypomethylation in human hepatocellular carcinomas correlates with shorter overall survival and CIMP phenotype. *PLoS One* **14**, 1–14 (2019). 10.1371/journal.pone.0216374 PMC6502450 31059558

[32] Zheng, Y. et al. DNA methylation of individual repetitive elements in hepatitis C virus infection-induced hepatocellular carcinoma. *Clin. Epigenet.* **11**, 1–13 (2019). 10.1186/s13148-019-0733-y PMC6802191 31639042

[33] Li, F. Y. et al. The co-occurrence of chronic Hepatitis B and fibrosis is associated with a decrease in hepatic global DNA methylation levels in patients with non-alcoholic fatty liver disease. *Front. Genet.* **12**, 1–9 (2021). 10.3389/fgene.2021.671552 PMC8318039 34335686

[34] Udomsinprasert, W. et al. Global DNA hypomethylation of Alu and LINE-1 transposable elements as an epigenetic biomarker of anti-tuberculosis drug-induced liver injury. *Emerg. Microbes Infect.* **10**, 1862–1872 (2021). 34467830 10.1080/22221751.2021.1976079 PMC8451674

[35] Huang, J., Wang, Y., Guo, Y. & Sun, S. Down-regulated microRNA-152 induces aberrant DNA methylation in hepatitis B virus-related hepatocellular carcinoma by targeting DNA methyltransferase 1. *Hepatology* **52**, 60–70 (2010). 20578129 10.1002/hep.23660

[36] Dahlhoff, C. et al. Hepatic methionine homeostasis is conserved in C57BL/6N mice on high-fat diet despite major changes in hepatic one-carbon metabolism. *PLoS One* **8**, 1–13 (2013). 10.1371/journal.pone.0057387 PMC3589430 23472083

[37] Lee, S. M. et al. HBx induces hypomethylation of distal intragenic CpG islands required for active expression of developmental regulators. *Proc. Natl. Acad. Sci. USA* **111**, 9555–9560 (2014). 24941955 10.1073/pnas.1400604111 PMC4084425

[38] Veloso Pereira, B. M., Charleaux de Ponte, M., Malavolta Luz, A. P. & Thieme, K. DNA methylation enzymes in the kidneys of male and female BTBR ob/ob mice. *Front. Endocrinol.* **14**, 1–10 (2023). 10.3389/fendo.2023.1167546 PMC10113614 37091852

[39] Sun, F. et al. TET3 boosts hepatocyte autophagy and impairs non-alcoholic fatty liver disease by increasing ENPP1 promoter hypomethylation. *Free Radic. Biol. Med.* **218**, 166–177 (2024). 38582229 10.1016/j.freeradbiomed.2024.04.207

[40] Sharma, A. et al. Detailed methylation map of LINE-1 5′-promoter region reveals hypomethylated CpG hotspots associated with tumor tissue specificity. *Mol. Genet. Genom. Med.* **7**, 3–7 (2019). 10.1002/mgg3.601 PMC6503062 30955237

[41] De Chiara, L. et al. Comparison of bisulfite pyrosequencing and methylation-specific qPCR for methylation assessment. *Int. J. Mol. Sci.* **21**, 1–13 (2020). 10.3390/ijms21239242 PMC7730915 33287451

[42] Hsueh, Y. M. et al. Associations among global long interspersed nuclear element-1 DNA methylation, metal exposure, and chronic kidney disease. *Arch. Toxicol.* **98**, 3127–3135 (2024). 38753188 10.1007/s00204-024-03780-9

[43] do Nascimento Gonçalves, N. et al. Distinct global DNA methylation and NF-κB expression profile of preimplantation biopsies from ideal and non-ideal kidneys. *J. Nephrol.* **35**, 1831–1840 (2022). 35524842 10.1007/s40620-022-01341-w

[44] Liao, L. M. et al. LINE-1 methylation levels in leukocyte DNA and risk of renal cell cancer. *PloS One* **6**, e27361 (2011). 22076155 10.1371/journal.pone.0027361 PMC3208631

[45] Sun, C. Y., Chang, S. C. & Wu, M. S. Suppression of Klotho expression by protein-bound uremic toxins is associated with increased DNA methyltransferase expression and DNA hypermethylation. *Kidney Int.* **81**, 640–650 (2012). 22237753 10.1038/ki.2011.445 PMC3306006

[46] Zeisberg, E. M. & Zeisberg, M. The role of promoter hypermethylation in fibroblast activation and fibrogenesis. *J. Pathol.* **229**, 264–273 (2013). 23097091 10.1002/path.4120

[47] Zhang, L. et al. DNA methyltransferase 1 may be a therapy target for attenuating diabetic nephropathy and podocyte injury. *Kidney Int.* **92**, 140–153 (2017). 28318634 10.1016/j.kint.2017.01.010

[48] Xiao, X. et al. Hypermethylation leads to the loss of HOXA5, resulting in JAG1 expression and NOTCH signaling contributing to kidney fibrosis. *Kidney Int.* **106**, 98–114 (2024). 38521405 10.1016/j.kint.2024.02.023 PMC12862895

[49] Yan, H. et al. Ten-eleven translocation methyl-cytosine dioxygenase 2 deficiency exacerbates renal ischemia-reperfusion injury. *Clin. Epigenet.* **12**, 1–8 (2020). 10.1186/s13148-020-00892-8 PMC7331250 32616016

[50] Bao, Y. et al. DNA demethylase Tet2 suppresses cisplatin-induced acute kidney injury. *Cell Death Discov*. **7**, 167 (2021). 10.1038/s41420-021-00528-7 PMC8257623 34226503

[51] Koh, E. S. et al. The protective effect of zebularine, an inhibitor of DNA methyltransferase, on renal tubulointerstitial inflammation and fibrosis. *Int. J. Mol. Sci.* **23**, 14045 (2022). 36430531 10.3390/ijms232214045 PMC9697081

[52] Akone, S. H. et al. Natural Products Impacting DNA Methyltransferases and Histone Deacetylases. *Front. Pharmacol.* **11**, 1–26 (2020). 32903500 10.3389/fphar.2020.00992 PMC7438611

[53] Qiu, W. et al. Kaempferol modulates DNA methylation and downregulates DNMT3B in bladder cancer. *Cell. Physiol. Biochem.* **41**, 1325–1335 (2017). 28278502 10.1159/000464435

[54] Kedhari Sundaram, M., Hussain, A., Haque, S., Raina, R. & Afroze, N. Quercetin modifies 5′CpG promoter methylation and reactivates various tumor suppressor genes by modulating epigenetic marks in human cervical cancer cells. *J. Cell. Biochem.* **120**, 18357–18369 (2019). 31172592 10.1002/jcb.29147

[55] Mukherjee, N., Kumar, A. P. & Ghosh, R. DNA methylation and flavonoids in genitourinary cancers. *Curr. Pharmacol. Rep.* **1**, 112–120 (2015). 26005633 10.1007/s40495-014-0004-8 PMC4437245

[56] Romagnolo, D. F., Donovan, M. G., Doetschman, T. C. & Selmin, O. I. N-6 linoleic acid induces epigenetics alterations associated with colonic inflammation and cancer. *Nutrients* **11**, 171 (2019). 10.3390/nu11010171 PMC6356359 30650553

[57] Remely, M. et al. Vitamin E modifies high-fat diet-induced increase of DNA strand breaks, and changes in expression and DNA methylation of DNMT1 and MLH1 in C57BL/6J male mice. *Nutrients* **9**, 1–20 (2017). 10.3390/nu9060607 PMC5490586 28613268

[58] Hernandes, L. C. et al. Caffeic acid and chlorogenic acid cytotoxicity, genotoxicity and impact on global DNA methylation in human leukemic cell lines. *Genet. Mol. Biol.* **43**, 1–8 (2020). 10.1590/1678-4685-GMB-2019-0347 PMC7350414 32644097

[59] Said, G. E. et al. Niacin based MOF as efficient nano-catalyst in the synthesis of some new benzothiazoles and benzimidazoles as anti-Alzheimer (AChE inhibitors). *J. Mol. Struct.* **1311**, 138462 (2024).

[60] Khatab, T. K., Kandil, E. M., Elsefy, D. E. & Mekabaty, A. One-pot multi-component catalytic synthesis of new 1H-pyrazole-1-carbothioamide derivatives with molecular docking studies as COX-2 inhibitors. *Biointerface Res. Appl. Chem.* **11**, 13779–13789 (2021).

[61] Zhang, Z. M. et al. Crystal structure of human DNA methyltransferase 1. *J. Mol. Biol.* **427**, 2520–2531 (2015). 26070743 10.1016/j.jmb.2015.06.001 PMC4520738

[62] Hu, L. et al. Crystal structure of TET2-DNA complex: insight into TET-mediated 5mC oxidation. *Cell* **155**, 1545–1555 (2013). 24315485 10.1016/j.cell.2013.11.020

[63] Al-Wasidi, A. et al. Cu-Vit B3 MOF solvothermal preparation, characterization and evaluation as HIV-1 RNA replication inhibitor. *J. Mol. Struct.* **1317**, 139120 (2024).

[64] El Makawy, A. I. et al. Exploration of tumor growth regression of quinoa and chia oil nanocapsules via the control of PIK3CA and MYC expression, anti-inflammation and cell proliferation inhibition, and their hepatorenal safety in rat breast cancer model. *Bull. Natl. Res. Cent*. **48**, 7 (2024).

[65] Boltz, V. F. et al. CpG methylation profiles of HIV-1 pro-viral DNA in individuals on ART. *Viruses* **13**, 799 (2021). 33946976 10.3390/v13050799 PMC8146454

